# Clinical and healthcare improvement through My Health Record usage and education in general practice (CHIME-GP): a study protocol for a cluster-randomised controlled trial

**DOI:** 10.1186/s13063-021-05438-8

**Published:** 2021-08-28

**Authors:** Andrew Bonney, Christine Metusela, Judy Mullan, Stephen Barnett, Joel Rhee, Conrad Kobel, Marijka Batterham

**Affiliations:** 1 University of Wollongong, Northfields Avenue, Wollongong, NSW 2522 Australia; 2 Medcast Pty Ltd., Sydney, NSW Australia

**Keywords:** General practice, Primary care, Education intervention, Health service utilisation

## Abstract

**Background:**

There is an international interest in whether improved primary care can lead to a more rational use of health resources. There is evidence that educational interventions can lead to improvements in the quality of rational prescribing and test ordering. A new national platform for shared medical records in Australia, My Health Record (MHR), poses new opportunities and challenges for system-wide implementation. This trial (CHIME-GP) will investigate whether components of a multifaceted education intervention in an Australian general practice setting on rational prescribing and investigation ordering leads to reductions in health-service utilisation and costs in the context of the use of a national digital health record system.

**Methods:**

The trial will be undertaken in Australian general practices. The aim of the research is to evaluate the effectiveness of components of a web-based educational intervention for general practitioners, regarding rational use of medicines, pathology and imaging in the context of the use of the MHR system. Our target is to recruit 120 general practitioners from urban and regional regions across Australia. We will use a mixed methods approach incorporating a three-arm pragmatic cluster randomised parallel trial and a prospective qualitative inquiry. The effect of each education component in each arm will be assessed, using the other two arms as controls. The evaluation will synthesise the results embedding qualitative pre/post interviews in the quantitative results to investigate implementation of the intervention, clinical behaviour change and mechanisms such as attitudes, that may influence change. The primary outcome will be an economic analysis of the cost per 100 consultations of selected prescriptions, pathology and radiology test ordering in the 6 months following the intervention compared with 6 months prior to the intervention. Secondary outcome measures include the rates per 100 consultations of selected prescriptions, pathology and radiology test ordering 6 months pre- and post-intervention, and comparison of knowledge assessment tests pre- and post-intervention.

**Discussion:**

The trial will produce robust health economic analyses on the evidence on educational intervention in reducing unnecessary prescribing, pathology and imaging ordering, in the context of MHR. In addition, the study will contribute to the evidence-base concerning the implementation of interventions to improve the quality of care in primary care practice.

**Trial registration:**

ClinicalTrials.gov ACTRN12620000010998. Registered on 09 January 2020 with the Australian New Zealand Clinical Trials Registry

## Administrative information

The order of the items has been modified to group similar items (see http://www.equator-network.org/reporting-guidelines/spirit-2013-statement-defining-standard-protocol-items-for-clinical-trials/).

**Table:** 

Title {1}	Clinical and Healthcare Improvement through My Health Record usage and Education in General Practice (CHIME-GP): A study protocol for a cluster-randomised controlled trial
Trial registration {2a and 2b}	Australian New Zealand Clinical Trials Registry ACTRN12620000010998. Registered on 09 January 2020
Protocol version {3}	Protocol Version 2.
Funding {4}	The Trial is funded by the Australian Digital Health Agency (ADHA). The University of Wollongong is contracted by Medcast Pty Ltd to provide the evaluation.
Author details {5a}	In order: Professor Andrew Bonney Dr Christine Metusela Associate Professor Judy Mullan Associate Professor Stephen Barnett Associate Professor Joel Rhee Dr Conrad Kobel Professor Marijka Batterham
Name and contact information for the trial sponsor {5b}	Australian Digital Health Agency 175 Liverpool Street Sydney NSW 2000
Role of sponsor {5c}	Other than requesting a randomised control-type design, the ADHA has no other role in the study design, data collection, management, analysis or interpretation of the data, writing of the report, decision to submit the report for publication or ultimate authority over any of these activities.

## Introduction

### Background and rationale {6a}

The My Health Record (MHR) system, established in 2012, is the national patient-controlled digital health record system administered by the Australian Digital Health Agency (ADHA). MHR is a secure online summary of patients’ health information, and all Australians have an MHR unless they chose to opt out before 31 January 2019. An individual can control what goes into their MHR and who is allowed to access it. They can choose to share their health information with their doctors, hospitals and other healthcare providers or they can also choose to permanently delete their records. One of the key aims of the MHR system is to support clinicians by helping to improve medication safety and reduce unnecessary test duplication [1]. Our randomised controlled trial (RCT) seeks to evaluate the effectiveness of a multifaceted educational package for clinicians regarding rational prescribing and ordering of pathology and radiology tests, in the context of the MHR system. The results of our RCT will inform education activities concerning MHR for clinicians.

The rational ordering of pathology and radiology, and appropriate prescription of medications, has significant implications for patient safety and efficient utilisation of healthcare resources and budgets. The literature demonstrates that over- and unnecessary use of prescriptions and test ordering is a major concern to health systems internationally [2, 3]. The unnecessary use of prescriptions and tests drives up health system costs, creates system-wide inefficiencies, and places patients at increased risk of harm [4]. There is international momentum to reduce unnecessary medicalisation in order to reduce these risks, supported by peak medical bodies in over 22 countries, including the UK, USA and Australia. Under the umbrella of ‘Choosing Wisely’, evidence-based, discipline and country-specific recommendations for reducing key unnecessary prescriptions and tests have been developed and made publicly available [5]. This study will compare medication prescribing and the ordering of pathology and radiology requests among GPs who receive an education intervention targeting one of these areas informed by the Australian National Prescribing Service (NPS) ‘Choosing Wisely’ recommendations [5] and other authoritative sources [6–11]. It is hypothesised that there will be a reduction in unnecessary medications and tests (pathology and radiology) being ordered among the GPs receiving the relevant education.

### Education interventions

Educational interventions have the potential for significant changes in clinical behaviour, with effect sizes in three recent systematic reviews of educational interventions showing a reduction in prescriptions by 15–20% [12], a reduction in diagnostic imaging by 10–25% [13] and a reduction in pathology ordering by 10–20% [3]. The most effective education-based interventions are those adopting a multifaceted approach, in particular practitioner education and feedback combined with systemic change [3]. Interventions that include the use of guidelines, audit, reflective practice (usually by way of clinical audit), workshops and academic detailing indicate the most benefit [2, 14–18]. In addition, GP alerting systems combined with practitioner education, including online tools and feedback have demonstrated to be beneficial in changing test ordering practices [19–21]. Similarly, clinical decision support technologies and drug usage advice have improved rational prescribing [22, 23]. To this end, primary care ‘groups’ have been shown to effectively allow practitioners to compare ordering and prescribing statistics and receive education [24].

According to the evidence therefore, the deployment of a multifaceted intervention in conjunction with the audit capacity facilitated by MHR has the potential to augment educational impact and change clinician behaviour. This is a logical step in patient safety and health records quality in Australia, whereby MHR provides a more accessible system-wide register of all medications and tests for patients and not just medications and tests within the patients’ practice health records. An exemplar of the synergies between education interventions and eHealth is demonstrated in the Extension for Community Healthcare Outcomes (ECHO) project which utilised telehealth, best practice protocols and multidisciplinary case-based learning to deliver sustained improved hepatitis C healthcare outcomes in an underserved population [25]. In our trial, the use of MHR will be presented as a resource to facilitate medicines and test ordering review within the online educational activities. However, even in the absence of education interventions, the implementation of eHealth records has proven to be beneficial in areas, such as smoking cessation through increased documentation [26]. In addition, eHealth data can be used to facilitate primary care audit and research [27] and can enable a reflective practice to explore prescribing and test ordering habits [28].

Pragmatic trials are viewed as a means of rigorously assessing the effectiveness of interventions in real-world settings to assist clinical or policy decision making [29]. It is important that policy makers and funders have high-quality evidence to support decisions, especially where there are significant clinical safety and financial implications. The extensive literature concerning the uptake of innovations in clinical practice demonstrates that the implementation process is complex, highly variable, non-linear and related to features of the innovation itself, such as the context into which the innovation is intended and the uptake of facilitatory supports [30–32].

Appreciation of the complexities of evaluating the implementation of change into health care systems has driven research approaches that have the capacity to describe not only the numeric end-result of the translation activities but also the important individual and system antecedents—what worked for whom, in what circumstances and why? [30, 33]. Such ‘realist’ approaches are important for policy makers to maximise the likelihood of achieving intended policy outcomes and enabling policies to be implemented in congruence with the context of the absorptive capacity of end-users and their environments. Thus, we have proposed a multi-method, inter-disciplinary realist-informed evaluation to maximise the utility of the evaluation findings for the ADHA.

### Rationale for extending phase I into phase II

This pragmatic trial is an extension of a pilot study (phase I) which reported promising outcomes from MHR educational intervention (ethics approval 2018/047) [34]. Phase I indicated that the intervention and use of MHR in clinical practice, based on general practitioner (GP) responses around intention-to-treat, demonstrated knowledge, skill and attitude changes among GP study participants with regards to evidence-based de-prescribing and ordering of pathology and diagnostic imaging tests. Phase I also demonstrated uptake of an ‘is this needed’ step in participants’ clinical reasoning and increased attention to reducing unnecessary healthcare expenditure. GPs reported, post-intervention, that they were more motivated to use MHR and were checking it more often, as well as feeling more confident about using it. In addition, there were significant behaviour changes regarding deprescribing. In particular, GPs reported that they discussed de-prescribing with patients more often, reduced rates of prescribing fluticasone (inhaled corticosteroid) and reduced prescribing of metformin and paracetamol/codeine combinations (among a subset of participants). There was also a significant reduction in test ordering for full blood count (FBC) and liver function tests (LFTs) from pre-intervention to post-intervention. The economic impact measured as part of the intervention indicated the potential for significant cost savings to the health system. Phase I however was limited by the following factors:

- Small sample size;
- Lack of controls for the educational components;
- Inability to access real-world outcomes to measure changes in clinician behaviour (using quantitative data) over time;
- Resultant restrictions on health economics analysis.

The current phase II study is designed to overcome the limitations of phase I.

### Objectives {7}

The objective of the study is to evaluate the effectiveness of a multifaceted educational intervention for Australian GPs. The educational intervention will be conducted by Medcast Pty Ltd. and is designed to support best-practice for prescribing, pathology and diagnostic imaging ordering in the context of the MHR system.

The trial will test the following primary hypothesis:

- The education intervention will result in a reduction in the cost per 100 consultations of specified prescriptions, pathology and radiology test ordering in the intervention versus control groups in the 6 months following the intervention compared with 6 months prior to the intervention.

Secondary hypotheses include that the intervention will result in the following:

- A reduction in the rate per 100 consultations of specified prescriptions in the intervention versus control groups in the 6 months prior to the intervention compared with 6 months following the intervention
- A reduction in the rate per 100 consultations of specified pathology test ordering in intervention versus control groups in the 6 months prior to the intervention compared with 6 months following the intervention
- A reduction in the rate per 100 consultations of specified radiology test ordering in the intervention versus control groups in 6 months prior to the intervention compared with 6 months following the intervention
- An improvement in knowledge assessment test scores in the intervention versus control groups in tests conducted prior to the intervention compared with following the intervention.

The specified prescriptions, pathology and radiology items are listed in Table 1.

**Table 1:** Target choosing wisely drugs and tests to inform the development of educational materials

List of choosing wisely based items	
**Rational prescribing**	
Proton pump inhibitors (PPIs)	
Diuretics	
Inhaled corticosteroids (ICS)	
Benzodiazepines	
Opiates	
Nonsteroidal anti-inflammatory drugs (NSAIDS)	
**Pathology ordering**	
Full blood count (FBC)	
Urea, creatinine and electrolytes	
Liver function test (LFT)	
Thyroid function test (TFT)	
Vitamin D	
Midstream urine (MSU)	
**Diagnostic imaging**	
Low back pain imaging - lumbosacral spine x-ray, lumbosacral spine CT scan and lumbosacral spine MRI scan	

We also aim to assess the health economic impact of the intervention and the contexts and mechanisms associated with the resulting outcomes.

### Trial design {8}

**Fig. 1:**
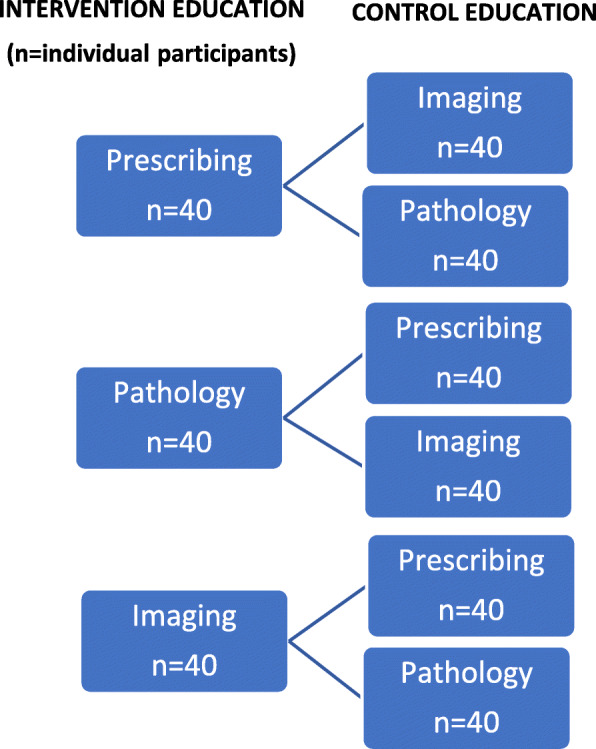
Study design

The design is a pragmatic cluster-randomised three-arm parallel trial and a prospective qualitative inquiry. The effect of the education topic intervention in each arm will be assessed, using the other two arms as controls. A schematic diagram of the study design is presented in Fig. 1.

The results will be synthesised in a mixed methods analysis embedding qualitative pre- and post-intervention interviews in the quantitative results.

Following ethics approval (HE2019/367), the trial was registered with the Australian New Zealand Clinical Trials Registry (ACTRN12620000010998).

### Methods: participants, interventions and outcomes

#### Study setting {9}

The study setting is among community-based primary care physicians (general practitioners - GPs) in Australia. Data will be collected from consenting GPs that participate in the online educational programme and assessments, the electronic health records generated by the GP participants and from telephone interviews with selected participants.

#### Eligibility criteria {10}

##### Inclusion criteria

- GP participants will be eligible for inclusion in the study if they hold an Australian Pharmaceutical Benefits Scheme (PBS) prescriber number and Medicare provider number; undertake clinical work at least 1 day per week in a primary care practice which has compatible electronic health records with PenCS data extraction tools installed and MHR access

##### Exclusion criteria

- Absence from clinical work for more than 8 weeks over the study period

#### Who will take informed consent? {26a}

Invitations and Participant Information Sheets (PIS) will be sent to eligible GP participants and their practices, via email or other electronic media, with two follow-up reminders. GPs who volunteer to be included in the study and their practices will be asked to complete and return a Consent Form to the UOW research team.

#### Additional consent provisions for collection and use of participant data and biological specimens {26b}

No additional consent provisions are required for the trial.

### Interventions

#### Explanation for the choice of comparators {6b}

Trial participants will be randomised to one of three parallel arms. Each arm will receive different topic content for prescribing, pathology ordering or imaging ordering as a standardised education process intervention. Each arm will act as controls for each other, for example, participants in the pathology arm will act as controls for the imaging and prescribing arms; participants in the imaging arm will act as controls for the pathology and prescribing arms; and participants in the prescribing arm will act as controls for the imaging and pathology arms. In this manner, the change in clinician behaviour associated with the education intervention for a given topic content arm can be compared against clinicians receiving the equivalent education process exposure but for different topic content in the other two arms.

#### Intervention description {11a}

##### Medcast support QI methodology

The standardised education process will be delivered by Medcast Pty Ltd. which uses best practice e-learning development, with a focus on social, interactive learning opportunities, known as the *Medcast Support QI methodology*. The educational theory underlying their work is ‘communities of practice’ [35], and ‘virtual communities of practice in General Practice’ [36] with tacit knowledge sharing (“know-how”), as well as formal, explicit knowledge sharing (‘know what’). Learning experiences are mostly case-based (scenario-based) learning and use a variety of modalities including webinars, self-paced learning, blended activities, quizzes and discussions. Two expert medical educators will develop the content for each study arm and also review each other’s content prior to the education being delivered.

##### Intervention

Medcast Pty Ltd. will develop and implement the education intervention which will focus on three topic streams running in parallel. The topic streams include a rational prescribing education intervention, a rational pathology-ordering education intervention and a rational diagnostic-imaging education intervention. All three topic streams will be in the context of the use of MHR in achieving quality improvement (QI) benefits and in integrating MHR into clinical practice. The ‘Choosing Wisely’ recommendations [5], an initiative of the National Prescribing Service Australia, will inform the education content, along with other sources of current evidence-based practice [6–11]. The Choosing Wisely recommendations, which are evidence-based, are supplied and endorsed by every peak medical and nursing body in Australia. The prescriptions and tests included in the study are specified a priori for the education sessions and then assessment. These same tests and prescriptions are assessed across all three arms of the trial 6 months pre- and post-intervention. The intervention pathology tests, imaging tests and prescription medication were chosen based on their frequency in general practice, cost to the PBS and potential for adverse outcomes in patients [13, 15, 16, 37].

**Fig. 2:**
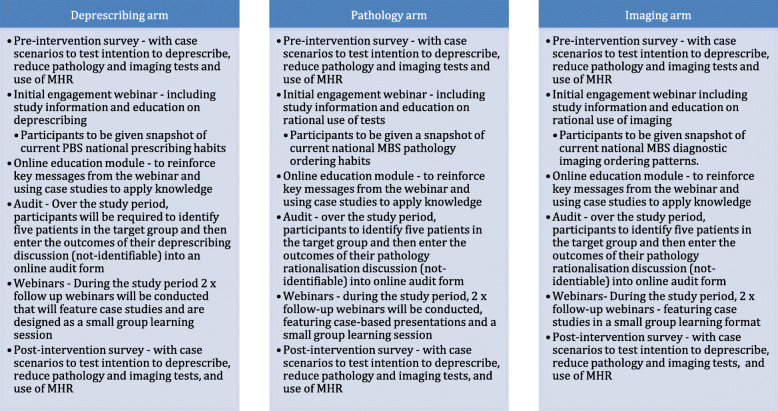
Description of education intervention activities for each arm

Each GP participant will be required to complete pre- and post-intervention assessments for all of the prescribing, pathology and imaging educational domains, as although participants are randomised into one arm of the trial, the other two arms act as controls. This will allow assessment of the knowledge and skills acquisition of the GP participants, attributable to the arm of the intervention they have been randomised into, in comparison with the other arms. See Fig. 2 for a comparison of the educational activities in each arm.

Due to COVID-19 [38] and the disruptions this has made to recruitment, we are holding two waves of the education intervention, 3 months apart, with up to 60 participants in each wave. This will enable us to recruit for a longer period of time and give us the opportunity to meet our recruitment target. It is envisaged that each wave of the educational intervention will be delivered over a period of 3 months and that the total estimated time commitment for participants is approximately 6 h (refer to Table 2 for the Medcast support QI methodology for the three arms of the intervention).

**Table 2:** Steps in the educational intervention

Description/activities	
**Study information and quiz/case scenarios (30 min)**	
Study information pack sent to participants.	
Participants to complete pre-intervention quiz with case scenarios to test intention-to-treat and current use of MHR.	
**Initial engagement webinar (45–60 min)**	
Evening webinar meeting facilitated by expert GPs.	
Study information to be presented to participants including key timelines.	
Opportunity for Q+A about the study process.	
Short didactic presentation introducing the topic relevant to their arm of the trial (safe use of medicines, pathology or radiology)	
Introduction to MHR, benefits and application.	
Arm-relevant case studies where the theory is applied in practice, plus case study examples.	
**Online learning module (60 min)**	
To include data of national, current trends in inappropriate prescribing and testing ordering, as relevant to each arm of the study.	
Information about MHR, the future of MHR and current uptake.	
Tips and tricks for engagement, barriers, algorithms that can be used.	
Information about where MHR can fit into practice—checking the record, look for duplicates, identifying medications prescribed or test results from elsewhere.	
A case study scenario demonstrating using MHR.	
Relevant case studies will be incorporated to apply the information delivered in the module.	
**Clinical audit - quality improvement (30–60 min)**	
Study participants will be required to complete a clinical audit (quality improvement) activity related to the study arm that they are in.	
The clinical audit activity will involve the collection and analysis of patient data with the intention to implement changes to clinical practice to improve patient outcomes.	
No identifying patient information will be collected or reported as part of the audit. The information will only be used by the GP for quality improvement activities.	
The information collected as part of the audit will be each participant’s reflection on the changes to clinical practice as a result of the study and audit activity.	
**Case preparation template for webinars (30 min each)**	
To prepare for the second and third case-based webinars study participants will be required to complete a case preparation template related to the study arm that they are in.	
**Case-based learning webinars/podcast (45–60 min each)**	
Webinars (two for each arm) will be held with the participants conducting the patient audits.	
The webinars will be facilitated by expert GPs and will provide participants with a virtual network to learn from via case-based learning.	
Short case presentations will be delivered during the study period and the participants will discuss the management.	
Participants will be encouraged to bring a case presentation from their clinical practice for discussion with the group.	
The webinars will be recorded and made available online or as a podcast.	
Webinar will be run during lunch time and evening to maximise the options for participation.	
**Post-intervention quiz/case scenarios (30 min)**	
Post-intervention case scenarios to assess intention-to-treat and use of MHR.	

#### Criteria for discontinuing or modifying allocated interventions {11b}

There are no specific criteria for discontinuation or modification of the intervention.

#### Strategies to improve adherence to interventions {11c}

Once enrolled in the education programme, participants will receive the standard prompts and reminders regarding the education intervention activities. This includes an email reminder to complete online activities prior to the commencement of the live webinars and once the live content has been delivered as well as the deadline to complete all activities. Email reminders are also sent via the zoom platform 1 week, 24 h, and 1 h prior to each live webinar, and a prompt sent once recordings of the live webinars are available online.

#### Relevant concomitant care permitted or prohibited during the trial {11d}

There are no restrictions placed upon participants for any other professional activity during the trial.

### Provisions for post-trial care {30}

As an educational activity, there are no provisions made for post-trial care, nor any participant harms expected.

### Outcomes {12}

#### Outcome measures

##### Primary outcome measure

- Difference in change in cost per 100 consultations of specified prescriptions, pathology and radiology test ordering in the intervention versus control groups for 6 months following the intervention compared with 6 months prior to the intervention.

Secondary outcome measures include the following:

- Difference in change in rate per 100 consultations of specified prescriptions in the intervention versus control groups for 6 months following the intervention compared with 6 months prior to the intervention.
- Difference in change in rate per 100 consultations of specified pathology test ordering in the intervention versus control groups for 6 months following the intervention compared with 6 months prior to the intervention.
- Difference in change in rate per 100 consultations of specified radiology test ordering in the intervention versus control groups for 6 months following the intervention compared with 6 months prior to the intervention.
- Difference in change in knowledge assessment test results in the intervention versus control groups, for tests conducted prior to the intervention compared with following the intervention.

To assess GPs’ clinical decision-making in the context of the MHR clinical system, the trial will include semi-structured interviews with approximately 30 participants before and after the intervention (approximately 10 in each arm), the final number depending on the sample at which data saturation has been reached.

#### Economic impact

Economic evaluation of the educational intervention will consider the joint cost and effects of strategies aimed at reducing potentially inappropriate prescribing, pathology and imaging, and report on intervention costs per session and individuals trained. Quantitative practice change evidence from GP data audit pre-post-intervention will be triangulated with GP self-reported assessment of the impact of training by Medcast. Within the study, a cost-effectiveness analysis will focus on observed intervention cost and cost offsets and, where appropriate, net incremental cost per reduction in reduced use of potentially inappropriate medicines, pathology and imaging.

### Participant timeline {13}

The time schedule of enrolment in the trial, data collection and intervention activities is shown in Table 3 below.

**Table 3:** CHIME-GP trial timeline

Task name	Start	Finish
**Participant recruitment**	**January 10, 2020**	**August 31, 2020**
o Invitations to participate will be communicated in recruitment waves via Medcast, PenCS, ADHA and UOW. Invitations will contain the study participant information sheet and consent forms for participants and practices as well as contact details of the research team.		
o Participants and practices expressing interest will send consent forms to the project officer at the University of Wollongong to be enrolled in the trial.		
**Participant randomisation**	**January 10, 2020**	**August 31, 2020**
o Randomisation of participants will occur after consent has been gained by participants and their practices. Each enrolled participant will be randomised into one of the three arms of the trial.		
**Data collection pre-intervention**	**January 10, 2020**	**September 10, 2020**
o A sample of consenting participants will be contacted directly by the research team to arrange qualitative interviews following randomisation. o GPs n = 30, 10 in each arm, approximately 30 min per interview		
o Pre-trial participant quizzes and case scenario activities will be collected by Medcast June–September 2020		
**Intervention period**	**June 2020**	**November 2020**
o Wave 1 webinars and online education modules June–August 2020		
o Wave 2 webinars and online education modules September–November 2020		
**Data collection post-intervention**	**September 1, 2020**	**May 31, 2021**
o The sample of consenting participants will be contacted directly by the research team to arrange follow-up qualitative interviews. o GPs *n* = 30, 10 in each arm, approximately 30 min per interview		
o Post-trial participant quizzes and case scenario activities will be collected by Medcast		
**Data extraction**	**June 1, 2021**	**June 1, 2021**
o Electronic Health Record data extraction for period November 2019–May 2021 o GPs n=40 in each arm, automated de-identified data extraction for 6 months prior to 6 months post-intervention		

### Sample size {14}

#### Sample size for quantitative data

The study is conservatively powered for testing significance in sub-group analyses (e.g. change in prescribing rates or change in pathology test ordering) with a 1:1 intervention: control allocation. As this is a three-arm trial, the final number of control cases in sub-group analyses will be twice those assumed in these calculations. A medium intervention effect (f2 = 0.15) is detectable at 80% power and α = 0.05 with 55 participants, in a two-arm trial (27.5 in each arm), analysed using a linear mixed model. The generally accepted practice level intra-cluster correlation coefficient (ICC) of GP behaviour is 0.05, and an average of three participating GPs per practice will be assumed. This results in a conservatively estimated design effect of 1.1. Thus, the target recruitment is a minimum of 31 participants in each of the three arms of the trial (n=93). To allow for 25% attrition, the study will aim to recruit 40 participants in each of the three arms (n=120).

#### Sample selection for qualitative data

On the consent form, GP participants will be asked to indicate whether they agree to be contacted for pre- and post-intervention interviews. A member of the research team invites participants, by their preferred contact method, to take part in 30 min pre- and post-interviews. The research team will use a purposive sampling approach (maximum diversity sampling) to derive the qualitative study sample, which will take into consideration participant age, sex, clinic size and location.

### Recruitment {15}

Medcast Pty Ltd., PenCS (a health analytics company providing data extraction services for the project), ADHA and the University of Wollongong (UOW) will send invitations to GPs in their pre-existing networks, and other known GP groups, inviting GPs from practices that already have PenCS software installed on their practice computing systems to participate (see also eligibility criteria section 10). The organisations sending out invitations will not have access to any of the email databases except their own.

The invitations will be sent to GPs and their practices, via email or other electronic media, with two follow-up reminders. If insufficient responses are received after sending two reminders, further invitations to new samples of GPs and practices will be sent in the above fashion (refer also to section 26A). Due to the 2020 global pandemic, COVID-19 [38], and its impact on recruitment into the study, we have extended the recruitment period to 8 months instead of the initial plan of 4 months, to enable the study to meet the recruitment target. In addition, two further reminder emails will be sent to all GP contacts until the recruitment target is met.

The invitations will have the Participant Information Sheets and Consent Forms attached. Interested GPs and their practices can respond by faxing or emailing consent forms to the UOW research team. Individual GPs as well as the responsible officer for practices will be required to complete consent forms (see also Section 27). Although consent will be obtained at the practice level and the individual GP level, only data relating to the consenting GPs will be extracted from the clinical information system in each practice.

### Assignment of interventions: allocation

#### Sequence generation {16a}

A stratified randomisation approach will be used to ensure a balance of practice sizes (≤ 5 GPs vs ≥ 6 GPs) and rurality. Randomisation will be conducted using the RALLOC command in STATA V15 or higher (StataCorp LLC., College Station Tx). While the study will be analysed at the level of individual participants, in order to minimise contamination of control groups, the education intervention will be randomised at a practice level.

#### Concealment mechanism {16b}

Randomisation will be carried out by the research team’s statisticians to one of the 3 groups. The statisticians will remain blinded as to the education intervention assigned to each group for analysis. The study participants will not be blinded as to allocation.

#### Implementation {16c}

After receipt of consent forms from practices and their associated GPs, a de-identified list will be compiled of practices and GPs including practice size (≤ 5 GPs vs ≥ 6 GPs) remoteness area of the practice (major city or other) [39] and number of GPs from the practices participating. No other information regarding the identity of the practice will be supplied. The statisticians (Batterham and Kobel) will apply a computerised stratified randomisation algorithm RALLOC (using STATA) to ensure a balanced allocation across each of the three arms of the trial according to practice size and remoteness area and number of participating GPs. GP participants and their practices will be randomised to one of the three topic streams (intervention arms) on a 1:1:1 basis. The statisticians will provide the project officer the randomisation sequence who will allocate the practices into the three trial arms on a first-come-first-serve basis. As outlined in the data management sections of the protocol, the statisticians will not have to access the coding sheets until after the final analysis is completed.

### Assignment of interventions: blinding

#### Who will be blinded {17a}

The statisticians will be blinded as to the intervention allocation for analysis. Trial participants, educators and other research team members will not be blinded.

#### Procedure for unblinding if needed {17b}

As no clinical treatments will be undertaken, the researchers do not believe there is a situation in which breaking the study codes will be required.

### Data collection and management

#### Plans for assessment and collection of outcomes {18a}

##### Quantitative data collection

PenCS will create an automatic data extraction macro that will extract relevant de-identified clinical data from the Electronic Health Records (EHRs) of the participating GPs at their participating practices. The de-identified clinical data will be encrypted and securely transferred directly from the GP practice server to a secure UOW data repository. Test quiz data will be collected by Medcast Pty Ltd. as part of the education intervention. These data will be securely transferred to the UOW data repository.

##### Qualitative data collection

It is planned to interview approximately 30 participants prior to and following the intervention, 10 from each of the three education intervention arms. The final number of participants will be determined by whether data saturation has been reached in the interviews. Data saturation will be assessed as occurring when no new information has been collected over two subsequent interviews. The pre- and post-intervention interviews will be conducted as semi-structured individual 30-min telephone interviews with practice GPs. The interview questions will be used to elicit perceptions and experiences of using MHR prior to the intervention; perceptions and experiences about the intervention; attitudes and behaviour toward rational prescribing and testing; and facilitators and barriers to achieving the expected outcomes of the educational intervention. The interviews will be audio-recorded, transcribed verbatim and coded to remove identifiers. The data collected are summarised in Table 4.

**Table 4:** Data collection schedule

Study phase	Methods	Participants	Purpose
**Phase 1: Before education intervention**	Qualitative pre-intervention interviews	GPs approx. 30 (n=10 in each arm), approx. 30 min per interview	To ascertain perceptions and attitudes of participants before the education session intervention. • Baseline context including the use of MHR and attitudes and behaviours regarding rational prescribing and testing • Key expectations of the educational intervention • Anticipated outcomes of the intervention • Expected facilitators of, and barriers to, achieving the expected outcomes
	Quantitative pre-intervention audit of rates of specified prescribing, pathology and imaging ordering and MHR access for 6 months prior to the intervention	GPs n=40 in each arm, automated de-identified data extraction	To describe baseline rates of selected prescribing, pathology and imaging
	Pre-intervention survey with case scenarios to test intention to prescribe/order and use of MHR	GPs n=40 in each arm, approx. 30 min per participant	To describe baseline knowledge and skills
	Quantitative project uptake data	GPs n=40 in each arm, automated data extraction	To assess GP engagement with the intervention module completions, webinar attendances and site logins will be collected by Medcast.
**Phase 2: After completion of education intervention**	Post-intervention knowledge assessment with case scenarios to test intention to prescribe/order and use of MHR	GPs n=40 in each arm, approx. 30 min per participant	To describe changes from baseline between arms in knowledge and skills
	Qualitative post-intervention interviews	GPs approx. 30 (n=10 in each arm), approx. 30 min per interview	To ascertain perceptions and attitudes of the participants after the education session intervention. • Post-intervention context including use of MHR and attitudes and behaviours regarding rational prescribing and testing • The degree to which key expectations of the educational intervention were met • The degree to which the anticipated outcomes of the intervention were met • Facilitators of, and barriers to, achieving the expected outcomes • What worked for whom, where and why?
	Quantitative Post-intervention audit of rates of selected prescribing, pathology and imaging ordering	GPs n=40 in each arm, automated de-identified data extraction	To describe changes from baseline rates of selected prescribing, pathology and imaging between arms of the trial
	Mixed methods synthesis	All	Synthesis and analysis to provide in-depth evaluation of the project.

### Plans to promote participant retention and complete follow-up {18b}

Once enrolled in the education programme, participants will receive the standard prompts and reminders regarding education activities that would normally occur when enrolled in an equivalent programme under non-trial conditions (outlined in Section 11C).

### Data management {19}

The UOW research team has created a Data Management Plan which addresses the separation of Medcast from data collection and analysis. Three data repositories will be established in CloudStor, a secure encrypted UOW approved cloud storage resource with secure password-only access and servers based in Australia. This will facilitate the separation of data from master code sheets and provide access to only those members of the research team who require access to the data. As part of this Data Management Plan, the Medcast team will only have access to the following data:

- GP knowledge Quiz data
- Project uptake data

Repository 1 will be shared between Medcast and UOW research team members. Coded knowledge quiz data will be stored in Repository 1 by Medcast. Following the intervention period, Medcast access to Repository 1 will be withdrawn. Repository 2 will be used to store the master coding sheet which links the GP participants’ identifying information to a six-digit study ID (coding the practice and the individual participant). This repository will also be used to store the randomisation allocation list. This repository will be kept separately and accessible only to the UOW Principal Investigator and project officer. Repository 3 will contain the coded, de-identified clinical audit electronic health record data. Via the PenCS software, de-identified electronic health record audit data will be transferred from the practices, coded by the participating clinician’s study ID, directly into this repository. This repository will also hold the coded, de-identified qualitative interview audio files and transcripts. This repository will be accessible only to the UOW research team members.

CloudStor, the cloud data platform to be used in the project, is administered by AARNet. UOW is a member of AARNet and CloudStor is a UOW approved research data storage option. CloudStor servers are located in Australia and data stored in CloudStor are encrypted and password protected. Ongoing access to the data will be limited to the UOW research team members involved in analysis and publications.

Hard copies of all consent forms will be stored in a locked filing cabinet in the Department of General Practice at UOW, or password-protected folder on CloudStor if in soft copy form. All data will be kept in accordance with UOW research data policies, stored for 5 years and thereafter permanently deleted.

### Confidentiality {27}

In order to maintain confidentiality and privacy, the researchers will establish secure data storage and management procedures.

A digital record of consenting GPs and practices will be available to Medcast and PenCS by the UOW research team by secure internet file transfer. This is needed as the Medcast research team requires access to identifying information of GPs and practices and identifiable participant knowledge quiz data in order to implement the education intervention and administer the professional development requirements, and PenCS requires identifying information of GPs and practices to perform the data extraction.

The UOW research team will allocate a six-digit study identifier to each consenting GP. The identifying information of the GPs and their study ID will be stored on a coding master-sheet kept securely and separately from all other study data.

The unit-level data from each practice is de-identified on-site by the PenCS software and coded according to the consenting GP generating the data. Only data generated by consenting GPs will be extracted.

The de-identified practice data will be transferred securely directly from the practice server to a secure UOW data repository (as is described in further detail below). Therefore, there is no risk that the data is at risk between points, as it is sent directly to UOW and not housed anywhere but the practice server and the UOW secure data repository.

All qualitative interviews will be digitally recorded and coded, then de-identified by the professional transcription service. Once quality checking of the coded audio files has been performed, the original audio files will be deleted. Participants can request removal of their identifying data at any time, and any re-identifiable data up until analysis is complete.

### Plans for collection, laboratory evaluation and storage of biological specimens for genetic or molecular analysis in this trial/future use {33}

No laboratory or biological specimens will be collected as part of this research.

### Statistical methods

#### Statistical methods for primary and secondary outcomes {20a}

The use of a randomised recruitment design and stratified randomisation of the intervention will reduce the potential for sampling bias. The statisticians will be blinded as to participant groups for analysis. Each of the data elements will be statistically analysed using linear mixed models test for between-group (intervention versus control) differences in the change in variables over the course of the study period accounting for any clustering. Where appropriate, analyses will control for demographic (age and sex) and practice environment (size, area-level socioeconomic disadvantage and rurality) variables and include MHR access rates as a covariate (the pre/post-evaluation of uploads of the MHR shared health summary).

#### Interim analyses {21b}

As this is an educational intervention, no interim analyses will be performed.

#### Methods for additional analyses (e.g. subgroup analyses) {20b}

The method for subgroup analyses will be the same as those for the primary analysis.

##### Qualitative analysis

We will use a framework analysis approach which will allow us to identify relationships and patterns across the qualitative data. Framework analysis has been widely used in health research [40, 41]. Importantly, framework analysis facilitates an ordered and organised way to compare and contrast data both across multiple cases and within individual cases. We will use the seven-step process proposed by Gale et al. [42] to undertake the framework analysis. These steps are [1] transcription, [2] familiarisation, [3] coding, [4] developing a working analytical framework, [5] applying the framework, [6] charting the data, and [7] interpreting the data. By taking this approach, we will be able to develop clusters of themes that facilitate both explanatory and descriptive conclusions directly relevant to our study objectives. We will utilise NVivo for coding and management of the qualitative data sets. A selection of the data will be double-coded to aid reliability. The researchers will employ reflexivity in the analyses to guard against bias arising from their roles and backgrounds.

### Methods in analysis to handle protocol non-adherence and any statistical methods to handle missing data {20c}

Data will be analysed on an intention-to-treat basis. That is, where available, data will still be included in the analysis where there has been non-adherence to the educational intervention or loss to follow-up. The planned mixed model statistical analysis is robust to missing data.

### Plans to give access to the full protocol, participant level-data and statistical code {31c}

The protocol is publicly available through this publication and the ANZCTR website.

### Oversight and monitoring

#### Composition of the coordinating Centre and trial steering committee {5d}

All Chief Investigators are members of the trial steering committee, which meets every 4 weeks. The steering committee’s remit includes data management. It is independent from the sponsor (ADHA). An operations group responsible for the day-to-day conduct of the trial reports to the steering committee and meets weekly.

#### Composition of the data monitoring committee, its role and reporting structure {21a}

Due to the low risk of harms arising from this educational intervention, the trial steering committee will act as the data monitoring committee. It is independent from the sponsor (ADHA).

#### Adverse event reporting and harms {22}

Any reports of potential harm from participating GPs will be investigated by the steering committee and, if appropriate, reported to the ethics committee.

#### Frequency and plans for auditing trial conduct {23}

There will not be any independent trial audit.

#### Plans for communicating important protocol amendments to relevant parties (e.g. trial participants, ethical committees) {25}

The research team will obtain ethics committee approval for all substantive trial amendments, including changes to recruitment, eligibility, the intervention or outcomes. Where relevant, substantive changes will be communicated to the sponsor (ADHA) via Medcast and to trial participants via the UOW research team.

#### Dissemination plans {31a}

Outcomes of the trial and recommendations will be communicated to the trial sponsor, ADHA, via a report and through presentations. The research group will also disseminate the trial findings through publications in peer-reviewed journals, conference presentations, professional meetings and plain language summaries on institutional websites.

## Discussion

This trial consists of a practice-based intervention implemented in the primary care setting. Through quantitative data collection and analyses, the trial is designed to test the effects of an education intervention to enable quality improvement in general practice. As part of the trial design, pre- and post-qualitative interviews are intended to contextualise the quantitative findings and provide insights into GP attitudes and behaviour toward rational use of medicines, pathology and imaging, and their perceptions about the success or otherwise of the intervention.

The research will evaluate the effectiveness of the educational intervention for GPs, regarding rational testing and prescribing in the context of the MHR system, in achieving the following objectives set by the ADHA, improving practitioner knowledge, changing practitioner behaviour, facilitating incorporation of clinical changes and technology usage into routine care, making meaningful improvements in clinical care and resulting in tangible economic benefits. Due to the challenges faced by conducting research during COVID-19, two waves of education intervention are planned to enable us to meet our recruitment target.

Exploring and influencing GP habits requires a pragmatic approach. System-based strategies such as protocol-based test ordering and the use of clinical guidelines have been shown to promote rational ordering and cost savings [43, 44]. Furthermore, patient engagement and health responsibility are enabled by MHR, encouraging shared decision-making and future possibilities of patient interventions [45]. Indeed, improvements in multimorbidity outcomes in primary care have resulted from better case planning and care coordination, further acknowledging the intended benefits of eHealth [46].

Educational interventions in general practice have the potential for significant savings to the Medicare Benefits Scheme (MBS) and Pharmaceutical Benefits Scheme (PBS)—medical services that are subsidised by the Australian government, even with moderate effect sizes of the interventions, with recent systematic reviews demonstrating a number of trials resulting in reductions in prescriptions [12], diagnostic imaging [13] and pathology ordering [3].

An appropriately designed, multifaceted education intervention, in the context of MHR, has the potential to improve quality of care and lower the burden of illness for patients while reducing costs from test duplication, polypharmacy and low-value test ordering. This pragmatic cluster-randomised three-arm parallel trial is adequately powered to quantify reductions in unnecessary prescribing, pathology and imaging ordering, and capable of producing robust health economic analyses.

### Limitations

There are potential limitations in the study described, including the potential for selection bias of more highly motivated clinicians. While the study design should allow for differentiation of the effects of each topic stream in the context of MHR use, there is no control arm for educational activities without MHR and MHR usage rates will be assessed on a pre-post trial basis.

### Trial status

The trial is completed. Recruitment commenced in January 2020 and concluded on August 31, 2020. The intervention was completed in November 2020 and data collection completed in May 2021. The study was still in the recruitment stage at the time of the protocol manuscript submission. Protocol version 2, January 9, 2020.

## Abbreviations

ADHA: Australian Digital Health Agency
ANZCTR: Australian New Zealand Clinical Trials Registry
EHRs: Electronic Health Records
FBC: Full blood count
GP: General practitioner
ICC: Intra-cluster correlation coefficient
LFTs: Liver function tests
MBS: Medicare Benefits Schedule
MHR: My Health Record
PBS: Pharmaceutical Benefits Scheme
PIS: Participant Information Sheet
QI: Quality improvement
RCT: Randomised controlled trial
UOW: University of Wollongong
